# Editorial: National representative dietary surveys and their importance for public health policies

**DOI:** 10.3389/fpubh.2023.1168396

**Published:** 2023-04-11

**Authors:** Valéria Troncoso Baltar, Diana Barbosa Cunha, Bartira Gorgulho, Bruna Kulik Hassan

**Affiliations:** ^1^Department of Epidemiology and Biostatistics, Institute of Collective Health, Fluminense Federal University, Niterói, Brazil; ^2^Department of Epidemiology, Hésio Cordeiro Institute of Social Medicine, Rio de Janeiro State University, Rio de Janeiro, Brazil; ^3^Department of Food and Nutrition, Federal University of Mato Grosso, Cuiabá, Brazil; ^4^ACT Health Promotion, Rio de Janeiro, Brazil

**Keywords:** national survey, diet, representative sample, public health policies, modifiable risk factor, National Dietary Surveys, food patterns

The relationship between poor diet and chronic diseases, morbidity and premature mortality has been extensively investigated. However, examining dietary habits is complex due to interactions between foods and nutrients. Moreover, valid data from national surveys are scarce and such analyses are important to support the formulation and evaluation of effectiveness of food and dietary guidelines, and public health policies in a more realistic way.

The aim of this Research Topic was to focus on investigating the diet quality or dietary patterns in national surveys at overall or meal levels, in order to add knowledge for public health policies. As well as to bring attention to the relevance of National Dietary Surveys to give support to public health policies, which justifies continuous national representative data collection. This volume gathers five papers considering National Dietary Surveys from the United States (US), India and Brazil. Specifically, the first article from the US analyzed diet in association with cancer mortality, and the second investigated the association of polyunsaturated fatty acids with female infertility. The third described malnutrition in India according to socio-demographic factors, and the other two, from Brazil, investigated trends in the volume of minimally and ultra-processed beverages purchased from 2003 to 2018 and beverage portion sizes consumption trends between 2008 and 2018.

Depiction of dietary patterns and investigating the role of diet in the etiology of non-communicable chronic diseases (NCDs) certainly constitute important challenges, given food consumption is one of the most difficult attributes to measure. As inadequate diet is one of the four main modifiable behavioral risk factors of NCDs (1), continuous and robust food consumption evaluation is crucial.

The paper by Chan et al. highlights the role of diet compared to physical activity on women's cancer mortality, which is the second leading cause of death in the US. Using a nationally representative sample of the US population, the Third US National Health and Nutrition Examination Survey (NHANES III) linked to the National Death Index, and the HEI dietary quality index, the authors were able to show that participants with a healthy diet had higher cancer survival. The authors found no difference in survival according to physical activity levels, probably due to the fact that all types of cancer were analyzed together. These results highlight the importance of a healthy diet that physicians and others who treat individuals with cancer must be aware of.

Wang et al. used data from three cycles of the nationally representative sample of the US population (NHANES) to investigate the effects of dietary intake of polyunsaturated fatty acids (PUFAs) on female infertility. They used the multiple source method to estimate the usual dietary PUFA intakes of all participants from two 24 h recalls using age, race and BMI as explanatory variables. They found that PUFA intake is only slightly associated with infertility, and they suggest that docosahexaenoic acid is protective, omega-6 fatty acids and total omega-6 fatty acids are risk factors for primary infertility in women. These interesting results suggest that women can improve fertility *via* daily diet.

The study from India used data from The Comprehensive National Nutritional Survey (CNN), the only nationally representative survey with nutritional and sociodemographic data in this country. Pandurangi et al. estimated stunting, thinness, overweight and obesity, and its predictors, among Indian children aged 10–19 years. They found a double burden of malnutrition with a very high prevalence of stunting, thinness and a high prevalence of overweight/obesity in Indian adolescents. Furthermore, they found an association of malnutrition with several typical characteristics observed in other Low-Middle-Income countries, such as gender, social class, education and wealth index families. The authors suggest that such associated factors can prioritize interventional strategies at the local level to eradicate malnutrition at the national level.

The first Brazilian study, from Cavalcante et al., described trends of beverage portion sizes consumption between 2008 and 2018, using data from the Brazilian Household Budget Surveys (HBS) of 2008–2009 and 2017–2018. The results point out that although there is a decrease in the frequency of beverage consumers between 2008 and 2018, the portion sizes of alcoholic beverages, flavored juices, caloric soft drinks, milk and milk substitutes, and fruit juices increases whereas coffee and tea portion sizes decrease. The authors refer to the portion size effect (PSE) phenome, responsible for favoring the consumption of increasingly high-calorie portions, suggesting that these results may be due to the larger portions offered in commercial establishments, favoring excessive beverages consumption.

The other Brazilian study also analyzed nationwide HBS data from 2002–2003, 2008–2009 and 2017–2018. Oliveira and Canella analyzed daily volume of beverages purchased per capita over the period and found a decrease in the acquisition of minimally processed beverages whilst a stability of ultra-processed beverages in 15 years and differed according to income. Regular soft drinks were the most purchased beverages in 2017–2018, whereas milk occupied this position as of 2002–2003. The authors concluded the importance of national representative surveys that can subsidize public policies to regulate ultra-processed beverages, such as taxation on ultra-processed beverages and restricted access in schools, in order to improve health.

This Research Topic grounds the idea of National Dietary Surveys as essential to give support to public health policies. Therefore, it brings attention to both general and specific population groups and gives subsidies for public health policies in the direction of curbing NCDs. Ultimately, national and representative surveys give essential information for monitoring and evaluation of the second and third Sustainable Development Goals, which propose the achievement of wellbeing for all people at all ages, the guarantee of food security and improved nutrition, and this Research Topic contributes to that.

## Author contributions

VB, DC, BG, and BH contributed as monitoring editors for this Research Topic. All authors have made a substantial, direct, and intellectual contribution to the work and approved it for publication.
